# White paper from a CTSA workshop series on special and underserved populations: Enhancing investigator readiness to conduct research involving LGBT populations

**DOI:** 10.1017/cts.2018.317

**Published:** 2018-10-01

**Authors:** Alicia K. Matthews, Kevin Rak, Emily Anderson, Wendy Bostwick, Jesus Ramirez-Valles, Raymond A. Ruiz, Kathryn Macapagal, Karriem S. Watson, Rohan D. Jeremiah, Amparo Castillo, Wendy Choure

**Affiliations:** 1 College of Nursing, University of Illinois at Chicago, Chicago, IL, USA; 2 Center for Clinical and Translational Science, University of Illinois at Chicago, Chicago, IL, USA; 3 Loyola University Chicago Stritch School of Medicine, Maywood, IL, USA; 4 School of Public Health, University of Illinois at Chicago, Chicago, IL, USA; 5 Feinberg School of Medicine, Northwestern University, Chicago, IL, USA; 6 University of Illinois Cancer Center, Chicago, IL, USA; 7 Jane Adams College of Social Work, University of Illinois at Chicago, Chicago, IL, USA

**Keywords:** LGBT, sexual and gender minorities, health inequalities, community engagement, special populations

## Abstract

Despite the significant health disparities experienced by lesbian, gay, bisexual, and transgender (LGBT) populations, few investigators affiliated with the National Institutes of Health-funded Clinical and Translational Science Award Programs are conducting research related to this underserved population. We provide recommendations shared during a half-day workshop aimed at increasing researcher readiness to conduct LGBT research. This workshop was presented as part of a series on conducting research with underserved populations offered by the Recruitment, Retention, and Community Engagement Program of the Center for Clinical and Translational Science at the University of Illinois at Chicago. Six LGBT health research experts provided focused presentations. The workshop presentations included a summary of significant health inequality issues, theoretical models relevant to research on LGBT health, best practices in measuring sexual orientation and gender identity, recommendations for recruitment and retention, a discussion of community engagement, and ethical considerations in conducting LGBT research. We provide a summary of recommendations to guide future research, training, and public policy related to LGBT health. The information can increase capacity among Clinical and Translational Science Award affiliated researchers in conducting research in this special population.

## Introduction

An explicit goal of the National Center for Advancing Translational Sciences is to promote the integration of special and underserved populations in translational research across the human lifespan [1]. Over the past decade, there have been several calls from federal agencies to increase the amount, variety, and quality of research on LGBT populations. For example, in 2011, the Institute of Medicine (IOM, now called Health and Medicine Division) published a report on LGBT health and made several recommendations intended to produce high quality data on LGBT populations. It calls for including measures of sexual orientation and gender identity in national epidemiological surveys, improving methods for collecting and analyzing data, increasing participation of LGBT individuals in research, and increasing researcher funding and training [2]. In 2012, the National Institutes of Health (NIH) issued a program announcement (PA-12-111) calling for specific research projects to address LGBT health disparities [3]. In 2016, the Director of the National Institute on Minority Health and Health Disparities designated the sexual and gender minority (SGM) community as a health disparity population, formally recognizing the disparities this population faces, and creating more funding opportunities for reducing these disparities [4]. In that same year, the 21st Century Cures Act called on the research community to develop valid and reliable methods for LGBT population research and addressing challenges with methodology [5]. Despite these important developments, investigator readiness to examine the issues related to sexual orientation and gender identity health remains limited [2].

**Fig. 1:**
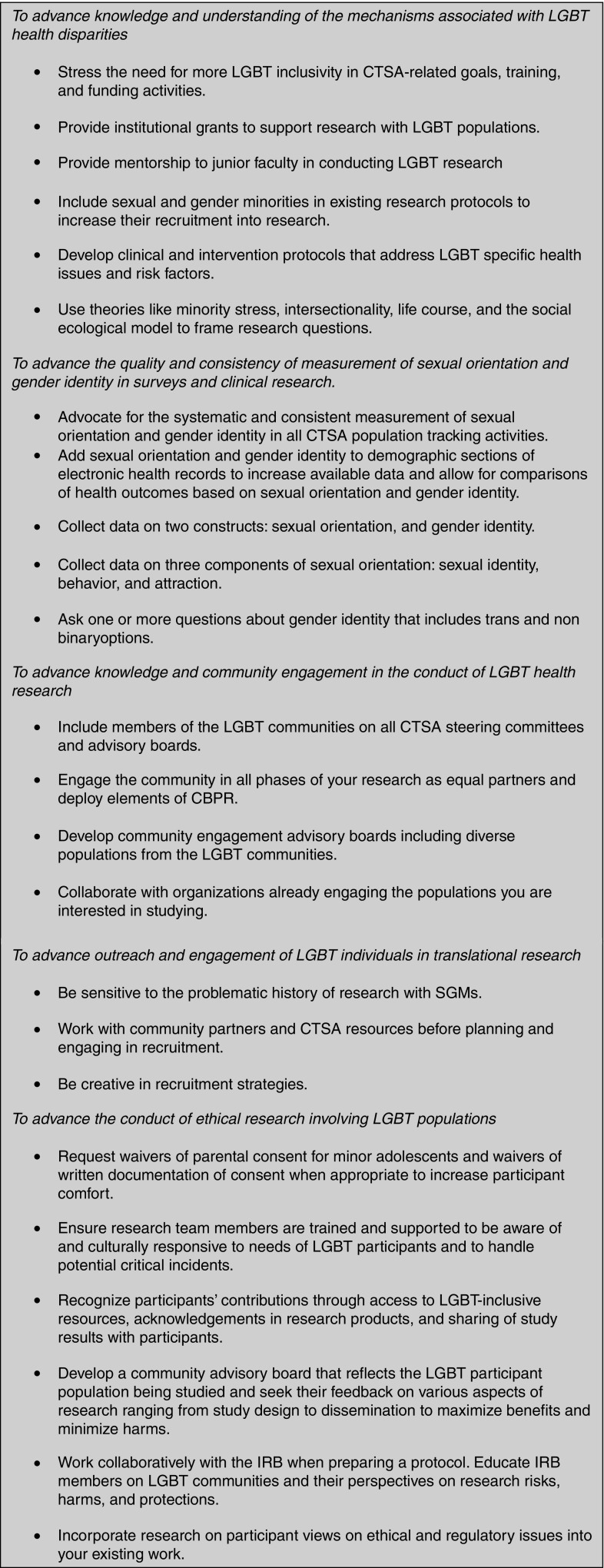
Recommendations for Clinical Science and Translational Award (CTSA) institutions and researchers. IRB, institutional review board; LGBT, lesbian, gay, bisexual, and transgender.

The NIH-funded Center for Clinical and Translational Science (CCTS) at the University of Illinois at Chicago seeks to improve population health with a particular emphasis on racial and ethnic minorities and other underserved populations. A key priority is to engage a broad range of stakeholders to increase the number and quality of studies that specifically address health disparity and special populations and to develop and disseminate resources to support this research. In October 2017, the Recruitment, Retention, and Community Engagement Program (RRCEP), a core program within CCTS, held its first Research Symposium on Special Populations. The 4-hour event, titled “Sexual and Gender Minority (SGM) Workshop: Research on the Health Disparities of LGBT Populations,” brought together a multi-disciplinary audience (n=60) of researchers, practitioners, community partners, students, and city government stakeholders to attend six presentations by LGBT health research experts. This article summarizes the key issues relevant to LGBT health research in order to enhance investigator readiness to engage LGBT communities in research by: emphasizing the importance of including LGBT individuals in research to address health disparities; discussing key theoretical and methodological issues relevant to conducting research involving LGBT populations; and offering recommendations for increasing the amount and quality of translational research involving LGBT populations. Recommendations for future research direction are also included (see Fig. 1). All presenters gave permission for inclusion of presented materials in this manuscript. All primary conference presenters were included as co-authors. Additional co-authors were members of the RRCEP team who provided direct input into the creation of the manuscript.

## LGBT Populations and Health Disparities

Sexual orientation is the enduring emotional, romantic, or sexual attraction to other people, and gender identity refers to one’s innermost concept of self as male, female, a blend of both, or neither. Importantly, one’s gender identity can be the same or different from their sex assigned at birth [6]. Consistent with the language used in the research literature, we will use the acronym LGBT to refer to SGM individuals while recognizing that these terms do not adequately reflect the heterogeneity of self-identifications or behaviors within these populations [7] (see https://www.lgbthealtheducation.org/wp-content/uploads/2018/03/Glossary-2018-English-update-1.pdf for a glossary of terms from the National LGBT Health Education Center).

Recent population-based studies have found that LGBT represent sizable minority groups in the United States. Although precise estimates are difficult to obtain, current estimates suggest that 4.1% of adults (approximately 10 million individuals) living in the US self-identify as LGBT [8]. Data from the Youth Risk Behavior Surveillance System, a nationally representative sample of 15,624 high school students, found that 8% identify as lesbian, gay, or bisexual [9]. A Williams Institute report suggests an additional 0.07% of youth ages 13 to 17 identify as transgender [10].

Emerging data from epidemiological studies have confirmed earlier descriptive research documenting elevated prevalence rates or risk factors for a range of physical and mental health conditions among LGBT populations including depression, cancer, asthma, cardiovascular disease, human immunodeficiency virus (HIV)/acquired immunodeficiency syndrome (AIDS), stroke, obesity, depression, and suicidality, to name a few [11]. These disparities in physical and mental health outcomes based on sexual orientation and gender identity have been observed across the life course, with LGBT adolescents and transgender individuals at particular risk [2]. In response to accumulating data, Healthy People 2020 for the first time identified sexual orientation as one of the five key demographic factors associated with poor health outcomes among Americans, paving the way for increased recognition of the health needs of this highly underserved population [12].

The factors associated with observed disparities among LGBT populations are complex and are likely influenced by the same sets of inter-related social, economic, and environmental factors that drive health disparities among other underserved populations [13]. However, unique risk factors experienced by sexual and gender minorities also exist. Exposure to bias-based stressors also extends to LGBT youth who experience higher rates of familial rejection, homelessness, school-based bullying, and school dropout compared with their heterosexual peers [14]. LGBT youth of color may be at an even higher risk for poor academic outcomes due to the corrosive influences of bullying and race-based discrimination [15]. These unique stressors experienced at the interpersonal, community, and institutional levels have direct implications for both short and long-term health risk behaviors [16]. In addition to the risk of poor health outcomes due to engagement in health risk behaviors, a strong evidential foundation supports the associations between chronic stress and inflammatory processes leading to chronic diseases including cancer [17].

Despite known health disparities, the inclusion of LGBT populations in clinical research as well as LGBT-specific research protocols is limited. Funding for LGBT focus research has also been limited. A recent review reported that LGBT studies account for just 0.5% of funded NIH studies, with the majority of these funded grants focused on issues related to the prevention and treatment of HIV/AIDS [18]. As a result, the majority of LGBT research unrelated to HIV/AIDS is hampered by small convenience samples, lack of comparison groups, and the absence of explanatory models [29]. Combined, the above factors have resulted in inadequate information by which to develop effective and evidence-based interventions to address the specific needs of LGBT populations.

Consistent with the recommendations of the Institutes of Medicine (2011), [2] the NIH, [4] and the 21st Century Cures Act, [5] additional high quality research is needed to better understand the origins of these disparities and to develop and test effective interventions to prevent and/or narrow observed differences in health risk behaviors and outcomes. Increased engagement of LGBT populations is within the mandate and scope of national Clinical Science and Translational Award (CTSA) programs.

## Theoretical Frameworks

Theoretical frameworks are formulated to explain, predict, and understand behavior, and to make transparent the assumptions underlying the study of a specific problem. Many of the existing frameworks for understanding individual-level predictors of health and health risk behaviors among adults in general can also be applied to research on LGBT populations (i.e., Transtheoretical Model of Behavioral Change [19]). Despite the utility of individual-level theories of human behavior, social factors including education, racial segregation, and poverty account for over a third of the total deaths in the United States each year [20]. In response, health disparity researchers are moving beyond the exclusive focus on individual-level predictors of risk to evaluate the influence of social determinants on persistent health inequalities. Similarly, there have been recent calls for the systematic study of the influence of social determinants (economic stability, neighborhood and physical environment, education, community and social context, and the health care system) on LGBT health inequalities [21].

In addition, the 2011 IOM report on LGBT research identified four conceptual frameworks viewed as highly applicable to understanding the mechanisms underlining observed health risk behaviors and outcomes among LGBT populations [2]. The most prominent of these frameworks is the Minority Stress Model [22]. A key assumption of this model is that LGBT-specific stress is unique and additive to general stressors that all people experience. Stress can come from both distal processes (clear cases of discrimination or violence) and proximal processes (perceptions of stigma) that impact the well-being of LGBT-identified individuals and communities [2]. In addition to direct experiences with discrimination, indicators of minority stress that have been examined among LGBT populations include concealment/fear of rejection, internalized homophobia, childhood adversity associated with gender nonconformity or sexual orientation, institutional discrimination, and other forms of discrimination not associated with sexual orientation (e.g., age, sex, and race/ ethnicity).

Intersectionality theory [23] posits that LGBT individuals must negotiate multiple complex social identities including their race/ethnicity and their gender identities which may lead to varied experiences, resources, adaptation strategies, and resiliency levels. Critical to this theory is that assertion that multiple group identities—each impacted by related systems of oppression—intersect to create a whole that is different from the component identities. All these levels have an impact on one’s health, and LGBT people can face stigma in their interaction with each one. Life course theory emphasizes how a person tends to revisit experiences they have had at earlier points in their life, and how events can have different impacts depending on when they happen in a person’s life [2]. This theory is useful in analyzing how sexual orientation and gender identity affect individuals at different stages of their lives. Finally, the Social Ecological Model [24] has also been proffered as a lens by which to understand the health and health outcomes of LGBT populations. This model considers the complex interplay between individual, relationship, community, and societal factors. It allows researchers to understand the range of factors that put people at risk for poor health or serve as protective factors for health and well-being.

## Measuring Sexual Orientation and Gender Identity

Historically, the availability of population-based epidemiological data on the health and health risk behaviors of LGBT individuals has been extremely limited. For example, the National Health Interview Survey only started asking about sexual orientation in 2013, and it does not inquire about gender identity [25]. Nevertheless, the inclusion of sexual orientation and gender identity as basic demographic measurements in research is increasingly commonplace and is considered best practice by a number of organizations, such as the American Psychological Association, and many others [26]. The systematic incorporation of sexual orientation and gender identity questions across health research studies is necessary to understand the full spectrum of health issues facing SGM populations, most especially due to consistent findings of a wide range of health disparities and inequities among LGBT groups [2].

### Asking about Sexual Orientation

The development and consistent use of validated measures of sexual and gender identity facilitates the collection of high quality research. Research has established that sexual orientation consists of at least three dimensions: identity, attraction, and behavior [27]. When possible, the recommendation is to assess all the three dimensions [28]. Different dimensions may be more or less associated with the specific health outcomes of interest, and asking about all three can help identify health risks and protective factors that may not be obvious. For example, in a study of mental health outcomes in the United States that considered associations across all the three dimensions of sexual orientation [29], the authors found that women who had sex with only women had significantly lower odds of lifetime mood disorders than other women. But, when assessing outcomes by sexual identity, women who identified as lesbian had significantly higher odds of a lifetime mood disorder compared with heterosexual women. Had the original survey not asked about sexual behavior in addition to identity, these differences would have been missed.

Recommendations regarding best practices for the wording of questions about sexual orientation in large surveys come primarily from a 2009 Williams Institute report [44]. (see Table 1). For sexual identity, the recommended question is: “Do you consider yourself to be: a) Heterosexual or straight; b) Gay or lesbian; or c) Bisexual?” This avoids using the term “sexual orientation,” which can confuse respondents. There is some debate as to whether or not to include additional response options for “other,” “don’t know” and/or “unsure,” as these responses would need to be coded or recoded after the fact, or in some instances, may be discarded altogether. However, in light of the dynamic and changing nature of sexual identity labels, including growing attention to those who self-identify as asexual [30], including the response option of “other (please specify),” would allow respondents to write-in the sexual identity label that they use and provides for the most succinct yet inclusive manner in which to ask about sexual orientation identity. The inclusion of the write-in option was recently endorsed in a white paper prepared by Federal Interagency Working Group on Improving Measurement of Sexual Orientation and Gender Identity in Federal Surveys [31].

**Table 1:** Recommended measures of sexual orientation and gender identity

Sexual identity	*Do you consider yourself to be:* (a) heterosexual or straight; (b) gay or lesbian; (c) bisexual; (d) something else? (please specify).
Sexual behavior	*In the past (time period, e.g. “year”) who have you had sex with?* (a) men only, (b) women only, (c) both men and women, (d) I have not had sex.
Sexual attraction	*Which of the following people do you find yourself feeling sexually attracted to?* (please check all that apply): (a) women/females, (b) men/males, (c) transgender women, (d) transgender men, (e) genderqueer/nonbinary individuals, (f) other (please specify).
Gender identity	Two-step approach 1. *What sex were you assigned at birth, on your original birth certificate?* (a) male (b) female. 2. *How do you describe yourself?* (check one): (a) male (b) female (c) transgender (d) do not identify as female, male, or transgender. One-Step Approach 1. *What is your current gender identity?* (check all that apply): (a) male (b) female (c) trans male/trans man (d) trans female/trans woman (e) genderqueer/gender non-conforming and/or (f) different identity (please state).

The recommended question to ask to assess the sexual behavior is: “In the past (time period, e.g., “year”) who have you had sex with? a) Men only, b) Women only, c) Both men and women, d) I have not had sex.” Rather than providing a definition of “sex” in the question itself, which has been found to create more confusion than clarity, the question leaves the decision up to the respondent as to how they define sex [44]. As noted, this question (and all questions that ask about behavior) should be temporally bound. The time period will depend upon the health issue being studied and other research-related factors. The recommended phrasing, however, does not allow the respondents to indicate transgender-identified or nonbinary individuals as sexual partners. As such, further question development is required to more fully capture the range of human sexualities. One possibility is adapting the measure from Herbenick *et al*. described below to sexual behavior. Until this or another question is psychometrically tested, however, we cannot give a clear recommendation on how to include trans and nonbinary individuals.

Although sexual attraction is often the dimension least likely to be captured in health surveys, it has particular utility when working with younger populations, who may still be forming their identity and/or may not be sexually active [32]. Until recently, sexual attraction questions used in large-scale surveys have only allowed respondents to note attraction to either women or men, and have not provided options that include attraction to persons who identify outside of those two categories. In order to capture a more complete picture of sexual attraction, we recommend the following question: “Which of the following people do you find yourself feeling sexually attracted to? Please check all that apply. Women/females, Men/males, Transgender women, Transgender men, Genderqueer/nonbinary individuals, and Other (please specify).” This is a newer measure reported by Herbenick *et al*. based on the National Survey of Sexual Health and Behavior that has not been tested as thoroughly as the identity and behavior measures; however, as noted, it improves on questions typically used (e.g., National Survey of Family Growth) by expanding options beyond just male and female (Herbenick, D., Dodge, B., Fu, T. C., Reece, M., Sanders, S., & Fortenberry, J. D. (2015). National Survey of Sexual Health and Behavior (data file). Unpublished raw data.)

Though the best practice is to include questions that capture all the three dimensions of sexual orientation in surveys, due to limited resources or concerns about survey length, investigators may want to include a single question. In this instance, we recommend asking about sexual identity. Sexual identity should be standard when assessing the demographic make-up of study samples or clinic intake forms, just like age, race, and education.

### Gender Identity

Gender identity is distinct from sexual orientation: it describes a person’s innate, deeply-felt psychological identification as a man, woman, or something else. Gender identity may or may not correspond to the person’s external body or the sex assigned at birth [33]. Those whose gender identity differs from their sex assigned at birth (e.g., identify as a woman, but assigned male at birth) are considered transgender. Those whose gender identity corresponds with their sex assigned at birth, e.g., identify as man, assigned male at birth, are cisgender.

Best practice recommendations from the Gender Identity in the US Surveillance Group [34] suggest what is known as the “two-step” approach to capturing gender identity. The first question asks about assigned sex at birth: “What sex were you assigned at birth, on your original birth certificate? Male, or Female”. The second asks about current gender identity: “How do you describe yourself? (check one) Male, Female, Transgender, or Do not identify as female, male, or transgender.” This question has been shown to have both high specificity (true negatives) and high sensitivity (true positives). Using the two-step approach, however, requires recoding by cross-referencing the two variables to create the categories of analysis. A promising one-question alternative is asking, “What is your current gender identity? (Check all that apply) Male, Female, Trans male/Trans man, Trans female/Trans woman, Genderqueer/Gender nonconforming, and/or Different identity (please state).” These recommended questions may continue to evolve as new literature emerges about transgender and nonbinary individuals (see Bauer *et al*. [35] for an in-depth assessment of transgender inclusive measures of sex and gender).

In sum, understanding the health needs of sexual and gender minorities requires, first and foremost, that our survey measures and data collection instruments are inclusive, and inquire about both sexual orientation and gender identity. Gender identity can be assessed through a single “check all that apply” question, or through a two-step process. When possible, surveys should inquire about sexual identity, attraction, and behavior. Minimally, sexual identity should be included as part of the standard battery of demographic questions.

## Community Engagement

Similar to other underserved and marginalized communities, researchers interested in focusing on LGBT populations may face difficulties in identifying and recruiting participants for research. Community engagement is a promising way to include more LGBT individuals in research. Community engagement can be understood as a strategic process aimed at establishing “collaboration between institutions of higher education and their larger communities (local, regional/state, national, and global) for the mutually beneficial exchange of knowledge and resources in a context of partnership and reciprocity [36] .” At its core, community engagement seeks to achieve equitable, meaningful, active community participation in all the phases of the research process [37] and highlights community strengths to accelerate improvements in health. Benefits of a community engaged approach to research include greater participation rates, increased external validity, decreased loss to follow-up, and the development of individual and community capacity [38]. Community engagement can also foster trust between communities and academic partners, particularly when there is a history of distrust [39]. The establishment of community advisory boards (CABs) is a proven strategy for increasing community engagement in research [40]. A CAB is usually a specialized entity assembled in a particular community for a particular research project; CABs tend to have homogeneous membership deriving from the topic of the search study [41]. A recent publication has described the development and evaluation of community engagement advisory boards for CTSAs; this process has applicability for engaging with the LGBT communities [42].

There are myriads of avenues for partnership and community engagement with organizations serving the needs of LGBT individuals and communities. Currently, there are numerous national LGBT organizations with branches or affiliations in the majority of all US states (www.lgbtcenters.org/LGBTCenters). Organizations also exist that are internationally focused (Centerlink), address disease-specific issues (National LGBT Center Network), are policy focused (Pride Action Tank and the Williams Institute), and professional organizations for LGBT health care providers (Gay and Lesbian Medical Association). In addition, many national organizations have participated in research and other outreach efforts to address LGBT health concerns (e.g., American Cancer Society). Incorporating the elements of community-based participatory research into LGBT health disparities research also provides an opportunity to have LGBT community members and stakeholders involved in all aspect of the research including prioritizing and developing research questions [43].

## Recruitment and Retention

A substantial amount of planning must go into sampling, recruitment, and retention of LGBT participants. Drawing a representative sample of LGBT individuals is expensive and difficult [44]. Most of the published research on this population has relied on convenience sampling, venue-based sampling, and random-digit-dialing methods [45]. Respondent-driven sampling (RDS) has emerged as an alternative for sampling hidden populations. RDS is a chain-referral method that helps assess and control selection bias, thus making it possible to derive population estimates [46]. RDS has been used successfully in studies involving gay and bisexual men and men who have sex with men [47]; however, recruitment outcomes associated with RDS approaches in studies involving young men who have sex with men [48] and lesbians [49] have been less successful.

Research teams can take several steps to foster trust with potential participants. Teams should be transparent about their motivations for undertaking the research project and what they hope to get out of it. They should also be aware of the reputation of the researcher and the institution, and be prepared to discuss potential participants’ concerns. Having at least some recruiters who share characteristics with the population being studied, such as LGBT status, can go a long way to improving recruitment. Although best practices have not been established for the recruitment and retention of LGBT individuals into research studies, successful strategies include clinic-based recruitment, active community outreach, passive community outreach, and social media. Clinic-based recruitment may include flyers and television announcements in the waiting room, letters to patients, and clinical staff referrals, particularly in settings that cater to the LGBT community. Active outreach involves study staff traveling to locations and events where eligible participants are likely to be and handing out stickers, palm cards, or small token items such as lip balm with study information. These materials are more likely to be successful than larger flyers or brochures at large events (e.g., the Pride Parade), where potential participants will not keep larger items. Passive outreach includes posting flyers in community locations, advertisements in printed publications, and messages to email lists. Photos in flyers can be tailored to include the racial/ethnic groups that are likely to see the flyers in any particular location

In addition to standard media coverage and social media apps like Facebook and Twitter, recent studies have used geo-social mobile applications “apps” like Grindr, Scruff, and Hornet to reach out to potential gay, bisexual, and transgender participants, and men who have sex with men participants [50]. These apps allow users to create a profile and utilize the phone’s location to introduce users in close physical proximity to each other. If users both users like each other’s picture and profile, they can chat. Though these very popular apps are often used for romantic pairings, researchers can use them to share study information. Recruiters can create profiles with study information, go to targeted locations, and use the app’s filters to prescreen users for potential participants. A standardized script can be used to guide interactions with potential participants [51]. Alternatively, potential participants can reach out to recruiters on the app. These social apps are useful for recruiting gay, bisexual, and transgender populations because users can avoid stigma and control when and how they choose to reveal their identity. In a study by Matthews *et al*., this method garnered a similar amount of participants as passive outreach, even though it ran for a much shorter time period and was less labor-intensive. Moving forward, geo-social apps represent a discreet way to reach and engage potential participants on their own terms.

## Ethical and Regulatory Considerations for Research including LGBT Populations

Conducting research that is representative and inclusive of LGBT populations requires an appreciation of certain ethical issues and the ability to navigate them successfully. Problems securing institutional review board (IRB) approval for studies involving LGBT populations have been documented [52]. Researchers have observed that subjective judgments about the appropriateness of or level of risk posed by research on ‘sensitive’ topics such as sex and sexuality, HIV status, and mental health, as well as problematic assumptions about LGBT populations (e.g., more prone to sexual risk behavior), may impact the decisions of IRB members, delay approvals, or prevent research from occurring altogether [52]. For IRBs less familiar with LGBT research protocols, collaborating with the IRB when proposing the study, educating the IRB about the potential risks and benefits of the study for the specific LGBT population under investigation, and documenting the team’s expertize in LGBT research can increase likelihood of study and waiver approvals [52].

LGBT populations historically have been mistreated in research (e.g., numerous studies testing harmful methods to alter sexual orientation and gender identity) [53]. In some cases, misrepresentation of research results has contributed to negative societal perceptions of LGBT populations [54]. For example, recently a controversial study asserting that children who were raised by LGBT couples had a variety of poor outcomes in adulthood was widely reported as evidence against allowing LGBT individuals legal rights to marry or have children [55]. Different researchers later reanalyzed the data and indicated the original findings were likely a result of measurement error and poor methodological choices [56], however, the initial study continues to be cited. These factors coupled with heightened concerns about privacy and confidentiality—particularly with regard to LGBT identity or potentially stigmatizing health conditions—can make potential participants wary of research involvement.

Mistrust in research may be greater in some subgroups of the LGBT community (e.g., transgender individuals, people of color) [57], and researcher transparency about motivations for undertaking the research project, project goals, and their expertize or identification with LGBT communities may foster trust and facilitate participation. In addition, LGBT participants may feel uncomfortable providing their name on a physical copy of a consent form due to privacy and confidentiality concerns. To address this, researchers can consider requesting a waiver of written documentation of consent from the IRB or making the study anonymous.

Certain aspects of a study may run the risk of exposing one’s LGBT identity or other marginalized or stigmatized aspects of their identity, behavior, or health to others. For example, advertisements that use terms or imagery reflective of the LGBT community may be seen by peers, coworkers, or family, and studies that occur in certain locations may inadvertently “out” a prospective participant (e.g., an LGBT community center) [58]. At the same time, such tailored recruitment materials and LGBT-friendly locations may be appealing for many other participant populations (e.g., out adults, those living in more progressive or urban areas). Thus, depending on the nature and context of the study investigators should consider the extent to which discretion is needed throughout their work. In addition, LGBT communities are small, even in large urban areas. For LGBT-identified researchers or staff, attempts should be made to avoid dual relationships with LGBT participants (i.e., they should not collect or handle identifiable data on a friend or colleague) by widening the scope of recruitment efforts beyond one’s immediate community [54].

In research with minors, researchers should consider whether parental permission is necessary and request waivers of parental permission for studies that pose no more than a minimal risk of harm. Requiring parental permission may require youth to disclose their LGBT identity to their parents for the first time, especially if it is clear from the recruitment and informed consent materials that the study is specifically for LGBT populations [59]. This is problematic, as youth’s disclosure of LGBT identity to their parents can often place them at risk of experiencing victimization, abuse, and rejection [60]. Requiring parental permission reduces LGBT youth’s willingness to participate in research, which biases samples toward youth who are out, have affirming relationships with their families, or do not engage in high risk behaviors [59], which in turn can bias research findings [61]. Studies have found that parents appreciate the potential risks and are generally supportive of waivers of parental permission for research on LGBT youth [62]. In the absence of parental permission, researchers can implement protections such as peer advocates or assessments of understanding.

Although ethical issues in LGBT research have long been documented in case studies and reports [54], only recently have studies begun to generate empirical evidence on this topic. Opportunities for further research exist, such as ethical issues related to research on gender-affirming interventions among transgender children and adolescents, data sharing of potentially sensitive information such as HIV status or sexual identity, or ethical issues in the use of constantly evolving social media and technologies to advertise, recruit for, and conduct research. Investigators are encouraged to incorporate research relevant to ethical issues into their larger projects—for example, adding questions about comfort or discomfort with novel study procedures or topics, participants’ opinions on adequate protections, or reasons for nonparticipation during the screening process—and to publish the results to advance evidence-based ethical practices for research on LGBT populations.

## Conclusion

Beyond the work of individual researchers, institutional support can facilitate better LGBT involvement and community engagement. Institutional leaders set the tone: if they stress the need for more inclusivity, it creates a culture where that is more likely to happen. Institutional grants provide an opportunity to support research with LGBT populations. Adding sexual orientation and gender identity to medical records can increase available data and allow for comparisons. Senior faculty have an important role in mentoring junior faculty to do LGBT research more effectively. Similarly, CTSA programs can help researchers develop the tools they need to conduct LGBT research. These steps will help develop the research needed to reduce LGBT health disparities. Researchers can further consult the Target Populations Toolkit, LGBT Toolkit developed by the RRCEP of the CCTS at the University of Illinois at Chicago, which is available at http://www.ccts.uic.edu/content/lgbt.
